# Reactivity to fearful expressions of familiar and unfamiliar people in children with autism: an eye-tracking pupillometry study

**DOI:** 10.1186/1866-1955-6-14

**Published:** 2014-05-31

**Authors:** Heather J Nuske, Giacomo Vivanti, Cheryl Dissanayake

**Affiliations:** 1 Olga Tennison Autism Research Centre, School of Psychological Science, La Trobe University, Melbourne, VIC, Australia; 2 The Victoria Autism Specific Early Learning and Care Centre, La Trobe University, Melbourne, VIC, Australia

**Keywords:** Autism, Emotion, Familiarity, Physiological reactivity, Pupillometry, Eye-tracking, Response latency

## Abstract

**Background:**

Individuals with autism are often reported to have difficulty with emotion processing. However, clinical and experimental data show that they are sensitive to familiarity; for example, they show normative attachment to familiar people, and have normative brain activity in response to familiar faces. To date, no study has measured their reactivity to the emotions of familiar vs. unfamiliar people. Thus, our aim was to determine whether individuals with autism would show normative reactivity to emotion in familiar people.

**Methods:**

Participants were 21 children with autism and 21 children with typical development, aged two to five years, matched on age and gender. The children observed videos of familiar people (their child-care teachers) and unfamiliar people expressing fear, whilst their visual attention and pupillary reactions were recorded (the latter as an index of emotional reactivity), using eye tracking technology.

**Results:**

The children with autism showed normative pupillary reactions (peak magnitude) to fear expressed by familiar people, but a reduced response to fear expressed by unfamiliar people. However, across familiarity conditions, the children with autism had longer latency peak responses than the typically developing children. This pattern of findings was independent of cognitive factors or visual attention as visual attention by group was not related to familiarity condition. The children with autism had reduced visual attention to neutral faces; however, on fearful faces there were no group differences. Abnormalities in pupillary reactivity in the autism group were related to less prosocial behaviour and more severe play and communication deficits.

**Conclusions:**

Children with autism were less atypical in their responses to fearful expressions of people they know, arguing against a pervasive emotional impairment in autism, but rather one that may be mediated by familiarity.

## Background

Emotional communication is one of the threads in the fabric of life that weaves together human relationships [1,2]. Individuals with Autism Spectrum Disorders (ASDs), a group of neurodevelopmental disorders defined by social-communicative difficulties and behavioural rigidity [3], have difficulty with the processing of emotions (for review, see [4]). For example, they have difficulty recognising subtle and complex emotional expressions [5,6], and a reduced physiological responses to emotion in others [7-9]. Despite the immense body of work on emotions in ASD to date (over 200 studies), nearly all studies have investigated the processing of emotion expressed by people who are unfamiliar to the participants [4]. Thus, an important question remains regarding whether individuals with ASD also have difficulty with the processing of emotion expressed by people who are familiar to them.

### Reactivity to the emotions of familiar vs. unfamiliar people

The differentiation in reactions to people based on familiarity is foundational for the organisation of social-emotional life [10,11]. Familiarity and prior affective contact with a person determines the personal relevance of their emotions; for example, a big smile from an old friend is not processed in the same way as a big smile from an unfamiliar person. In typical development, evidence from social and developmental psychology indicates that the processing of emotions of familiar people is easier, faster and achieved earlier in development, than the processing of emotions of unfamiliar people [12-19]. Moreover, recent evidence suggests that even at 12 months, infants have similar brain activation patterns to adults during the processing of emotion in familiar people [20]. Furthermore, typically developing individuals show more empathy towards familiar than unfamiliar people (for review, see [21]).

The only published study to date that has specifically contrasted emotion processing of familiar vs. unfamiliar people in participants with ASD has shown that children with ASD, like typically developing children, are more empathic towards caregivers than to unfamiliar people [22]. However, a recent study by Fox and colleagues [23] on infants with a high- and low-risk for ASD (that is, children who did, and did not have an older sibling with ASD, respectively), found that high-risk infants did not show differentiated brain responses to happy expressions of familiar vs. unfamiliar people, as did the low-risk infants. Thus, more research is needed to determine whether individuals with ASD react to the emotions of familiar people in a normative way, and whether this is related to their everyday empathic functioning. This information is crucial for advancing our understanding of emotion processing in ASD; if normative processing of emotions in familiar people is found, then the neural architecture for emotion processing may be functional in ASD, but may require ‘bootstrapping’ from other neural circuits, such as those involved in the processing of familiar persons (for review, see [24,25]).

### Neural responses to familiar vs. unfamiliar people

Differential neural responses to emotions expressed by familiar vs. unfamiliar people rely on early emerging capacities to distinguish between people who are known vs. people who are not. Indeed, newborn infants show different behavioural responses to (emotionally neutral) familiar and unfamiliar people [26-28]. A developmental transition in responses to familiar vs. unfamiliar people is apparent in typical development, whereby very young infants (six to nine months old) show greater brain responses to familiar (neutral) faces than to unfamiliar (neutral) faces [29-31], in 18- to 24-month-old children this differentiation is marginal, it disappears in 24- to 45-month-olds and re-emerges in the opposite direction in 3- to 6-year-olds [32-35]. Thus, during the first years of life there seems to be a shift from a greater neural response to familiar faces to a greater neural response to unfamiliar ones.

Research on the processing of familiar vs. unfamiliar faces in ASD suggests that children, adolescents and adults process (emotionally neutral) familiar faces using typical neural networks, despite atypical brain activation during the processing of (emotionally neutral) unfamiliar faces [36-38]. Furthermore, whilst typically developing preschoolers show greater neural responses to unfamiliar (neutral) faces, relative to familiar (neutral) faces, pre-schoolers with ASD do not show this pattern of responses [33] (but see [39]). Together, these findings suggest neural abnormalities in the processing of unfamiliar, but not familiar, but not familiar persons, in those with ASD. This evidence is consistent with data suggesting that children with ASD are able to form attachments with their caregivers [40-42], along with findings of abnormalities in approach behaviour towards unfamiliar people [43-45].

### Autonomic responses to familiar vs. unfamiliar people

Other than by measuring neural responses, responses to familiar vs. unfamiliar people may be captured by measuring autonomic responses. Certain autonomic nervous system (ANS) or physiological indicators signify only the sympathetic or parasympathetic branch of the ANS, such as skin conductance responses and respiratory sinus arrhythmia (RSA), respectively; other measures are indicators of both branches, such as heart rate and pupil size [46]. Only one study has examined autonomic responses to familiar vs. unfamiliar people in autism, finding that school-aged children with autism had decreased RSA to unfamiliar people reading a story, compared to typically developing children, with no group differences in RSA to familiar people reading a story. However, to date, no study has measured autonomic responses to the emotion of familiar vs. unfamiliar people in individuals with ASD.

### Timing of emotional responses in autism

Aside from the magnitude of one’s emotional responses, the timing of these responses plays an important role in everyday emotional reactivity and social reciprocity. For example, a delayed social smile from someone carries with it a different meaning than a social smile that is temporally contingent (upon the initial smile). In ASD, atypical emotional reactivity has been documented in studies focused on latency/timing of response to faces and other stimuli. Research on the processing of unfamiliar faces in individuals with ASD has identified slower event-related potentials (ERPs) to faces [47] and to emotional facial expressions [48], as well as slower emotion recognition [49,50] and delayed facial reactivity to emotions [51]. Importantly, shorter response latency to emotion in unfamiliar people has been associated with more empathic behaviour in young children with ASD [52], and more accurate emotion recognition in children and adolescents with ASD [53].

Few studies have reported on latency of response to familiar people in individuals with ASD. Dawson *et al*. [33] found no group differences in ERP latency to neutral facial expressions of familiar people in pre-schoolers with ASD compared to controls; however, there were also no group differences in ERP latency to unfamiliar people in this study. Key and Stone [54] studied ERP latency of response to expressively neutral familiar faces in infants with a high- and low-risk for ASD, and found that whilst low-risk children showed a longer latency response to unfamiliar faces (compared to familiar faces), high-risk children did not differentiate on the basis of familiarity. As no study has examined how individuals with ASD react to emotion in familiar people, it is also unknown if the timing of their response to emotion in familiar people is normative.

### Measuring emotional responses with eye-tracking pupillometry

As emotional responses can be captured with a variety of neural and autonomic indicators, it is important to consider the advantages of a given measure over other measures. The recording of pupillary reactivity, a reliable indicator of emotional arousal [55,56], using eye-tracking pupillometry (which measures pupillary responses with sensors in a computer-like monitor), offers three main advantages. First, eye-tracking pupillometry is non-invasive, and thus circumvents the issues surrounding the application of electrodes (for measuring ERPs, skin conductance responses or heart-rate), as this may in itself cause elevated arousal in these individuals due to common tactile sensitivities [57].

Second, as the measurement of neural and autonomic responses is sensitive to motion and individuals with ASD often have difficulty in following instructions and staying still for prolonged periods of time, movement-related artefacts are common in such data [58,59]. However, advanced eye-tracking pupillometry systems are not susceptible to these artefacts (explained further in the Apparatus section).

Third, this technique may be easily combined with traditional eye-tracking parameters (that is, for gaze analysis). This is important, as attention to the emotional stimuli as well as gaze patterns may be measured, and relationships between attention patterns and emotional reactivity can be explored. For example, one study found no group differences in pupillary response to facial emotions; however, in the ASD group only visual attention to the mouth region was related to the pupillary response [60]. Thus, this technology allows for an analysis of the relationship between attention to emotions and emotional reactivity, which is not available to neurophysiological and physiological systems that are not combined with eye tracking.

The use of eye-tracking pupillometry is fast gaining momentum in the study of emotion and face processing in ASD. One study identified that individuals with ASD, unlike typically developing individuals, have a greater pupillary response to inverted, relative to upright neutral faces [61]. Another study found that unlike control participants, individuals with ASD do not have greater pupillary responses to smiling faces with direct eye gaze, compared to smiling faces with averted gaze [62]. Also, two recent studies have identified abnormal pupillary reactions to emotional facial and vocal expressions in individuals with ASD [8,63].

Studies using traditional eye-tracking parameters have shown that individuals with ASD use atypical face-scanning patterns, most notably, with less attention to the eye region [64-67]. Sterling and colleagues [68], examining familiar and unfamiliar (neutral) face-scanning, found less visual attention to the eye region in adults with ASD compared to typically developing adults, regardless of whether the stimulus face was familiar or unfamiliar. Thus, the face-scanning pattern of emotionally neutral familiar faces also seems to be atypical in ASD; however, it is not yet known if this applies to familiar faces expressing emotion.

### The current study

The aim in the current study was to determine whether preschool children with ASD react to emotion in familiar vs. unfamiliar people in a normative way. To do so, we measured the pupillary reactions (magnitude and latency) of children with ASD and age- and gender-matched typically developing children to the fearful expressions of familiar vs. unfamiliar people, using eye-tracking pupillometry. We chose to use fearful facial expressions as this emotion has been found to produce large, detectable neurophysiological and physiological responses [69,70]. Also, some research has indicated that individuals with ASD may have particular difficulty with recognising and reacting to fearful expressions of unfamiliar people [52,71-74], but it is unclear if this is also the case for fearful expressions of familiar people.

Based on the findings reviewed above, we predicted that children with ASD would show abnormal reactions to fearful expressions in unfamiliar people, but normative reactions to fearful expressions in familiar people, compared to the comparison children. Furthermore, we predicted that the children with ASD would have less visual attention to the eye region of neutral faces, across familiarity conditions. We also aimed to explore any group differences in visual attention to fearful faces, to examine whether everyday empathic behaviour was related to attention as well as to the magnitude and latency of reactivity in each group. We also investigated the relationship between emotional reactivity and autism severity.

## Methods

### Participants

Twenty-five children with ASD and 21 typically developing (TD) children, aged two to five years, participated in the study. However, four children in the ASD group were excluded due to low visual attention (<20%) to the stimuli, that is, <800 ms viewing time of the face region (Eye area of interest (AOI) + Mouth AOI) during the neutral or fear still frames of any of the videos (see Materials section). This resulted in 21 participants with ASD being included in subsequent analyses.

Participant characteristics are presented in Table 1. Both groups were recruited through a community childcare centre. The Mullen Scales of Early Learning was administered to all participants to measure cognitive ability (MSEL [75]). Following the recommendations of Dykens and Lense [76], our group was representative of the greater ASD population in terms of cognitive ability [77], with 66.7% low-functioning and 33.3% high-functioning (at a cut-off of 70 standard score for high-functioning [78]). Thus, as expected, the ASD group was lower in cognitive ability than the TD group (who all had a MSEL standard score in the normal range). Cognitive ability was, therefore, used as a covariate in the analyses, where appropriate.

**Table 1:** Participant characteristics

	***ASD group (N = 21)§***	***TD group (N = 21)***	***t-*** **value**	***P-*** **value**
Age (years): *M* (SD)	3.98 (1.05)	4.27 (0.60)	1.095	.28
Gender: M, F	18, 3	18, 3	-	-
MSEL, SS*: *M* (SD)	67.86 (23.53)	100.29 (16.41)	5.18	<.00
ADOS, SC†: *M* (SD)	13.81 (5.16)	-	-	-
ADOS, RRB††: *M* (SD)	3.48 (1.81)	-	-	-

*Mullen Scales of Early Learning standard score (Early Learning Composite), *†*Autism Diagnostic Observation Schedule, Social-Communication Algorithm Total, *††*Autism Diagnostic Observation Schedule, Restricted and Repetitive Behaviours Algorithm Total, § 21 children after exclusion of four children for low visual attention (<20%) to the stimuli. ASD, autism spectrum disorder; TD, typically developing

Diagnoses of the children with ASD were confirmed using the Autism Diagnostic Observation Schedule (ADOS; [79]) by expert clinicians with 15 children meeting criteria for Autistic Disorder and 6 meeting criteria for ASD. One participant was taking methylphenidate at the time of testing; however, as this participant was not an outlier on any dependent variable, and results were not altered with the exclusion of the participant’s data, he was retained in the sample. Exclusion criteria for the TD children were a history of autistic symptoms, as reported by their parents and the child-care staff. All participants, including the typically developing participants, were free from any other medical conditions, and had no visual, hearing or motor impairments. The research was approved by the La Trobe University Human Ethics Committee (Approval Number 11–052).

### Apparatus

**Figure 1:**
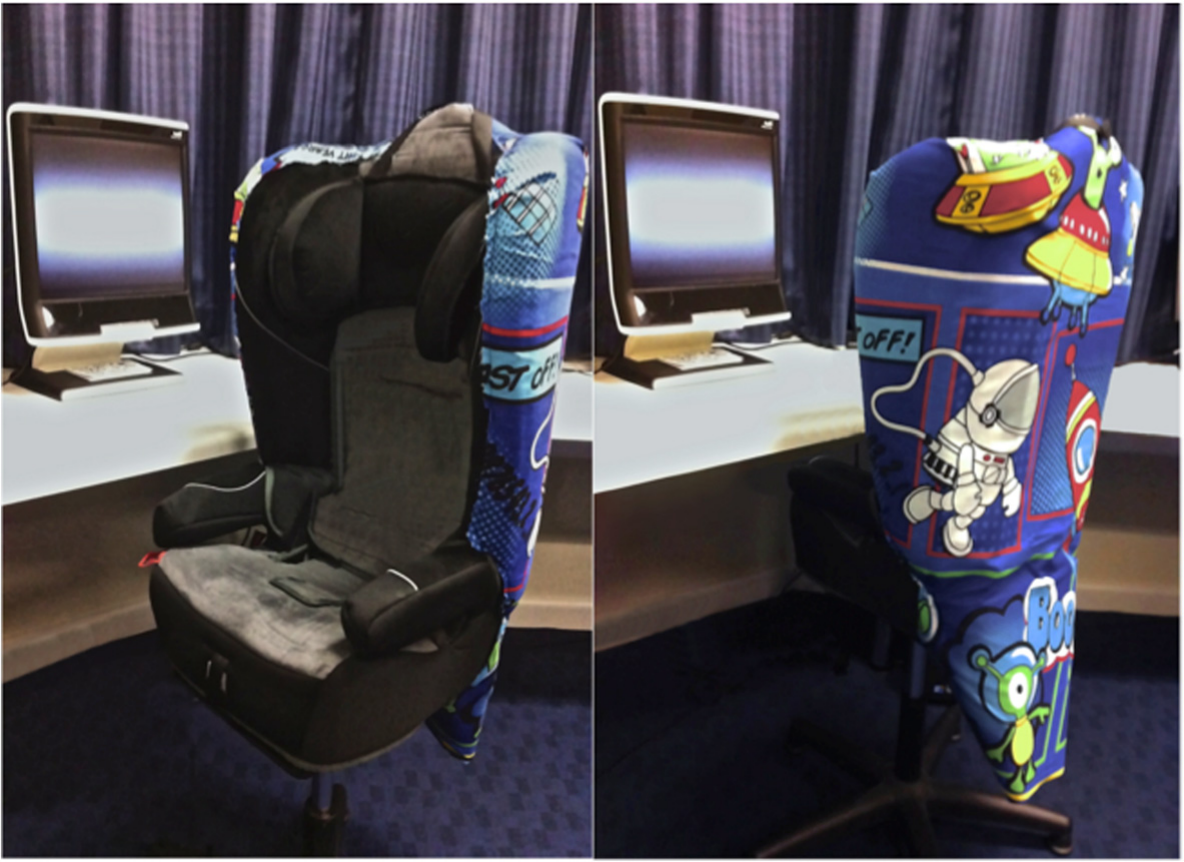
Experimental set-up.

A Tobii 120 binocular eye tracker and Tobii Studio software (version 3.0.3 Tobii, Stockholm, Sweden) was used to present stimuli and record gaze position and pupil diameter. This system presents stimuli on a computer-like monitor (see Figure 1) and does not require any equipment to be fastened onto the participant. Using multiple sensors, with bright and dark pupil tracking, a 3D model of the pupil (taking into account optical distortions from the cornea and lens) is built, allowing for both pupil diameter and distance to be measured at a sampling rate of 60 Hz (one sample every 16.67 ms). With this tracking technique movement-related artefacts are dealt with in two ways. Firstly, as pupil size is usually a function of distance (of the participant’s head to the monitor), the effect of head movements (perpendicular to the monitor) was eliminated from pupil diameter on a sample-to-sample basis by the Tobii system, using basic principles of trigonometry. Secondly, other head movements (that is, parallel to the monitor) were accurately tracked by the Tobii system (up to 25 cm per second). Brightness of the eye-tracking monitor (W: 34 cm × H: 27 cm) was set to 100%.

### Materials

#### Video filming

**Figure 2:**
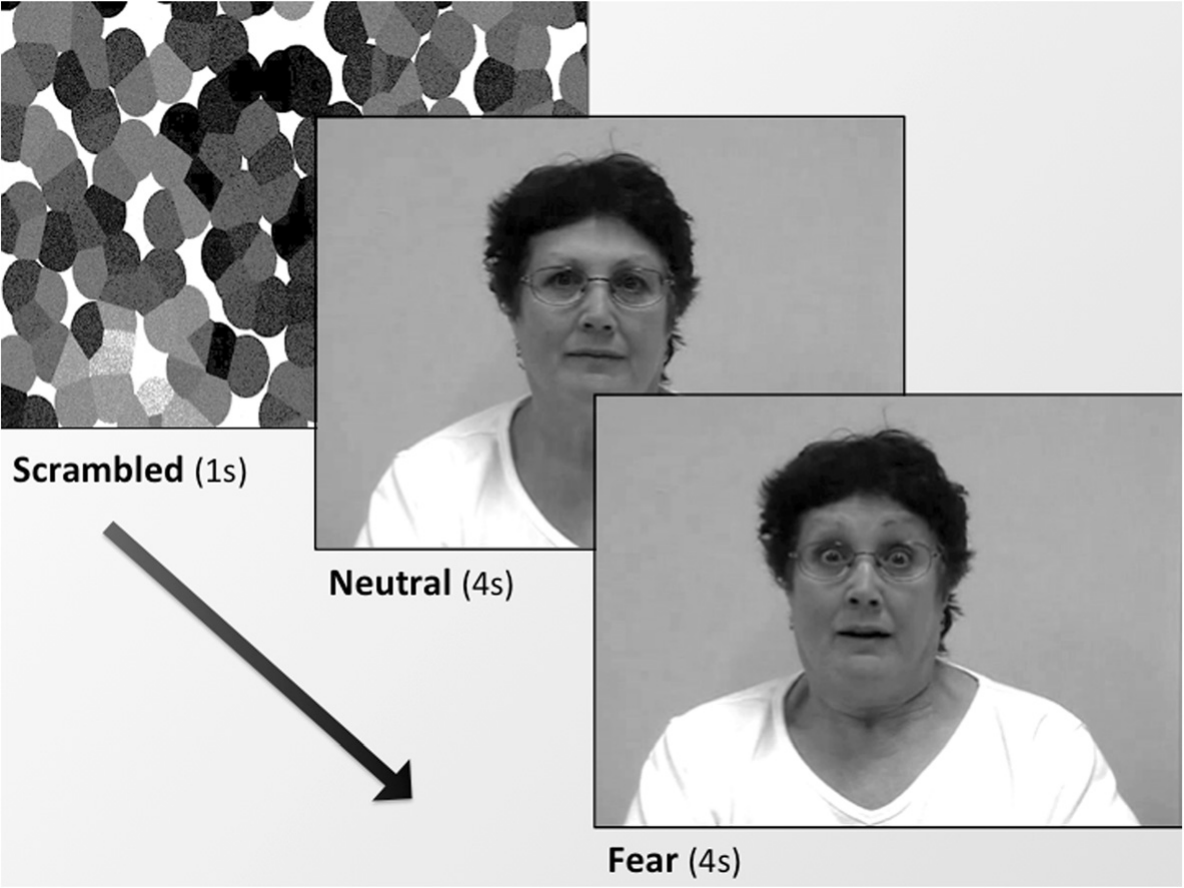
**Still frames taken from video stimuli.** The time between the Neutral and Fear frames varied slightly between ‘actor’ to 'actor' (*M* = 2.167 s, *SD* = 0.600 s).^e^

Stimuli were videos of people initially showing a neutral expression, which gradually changed into a prototypic fear expression (eyes widened, eyebrows lifted, mouth ajar; see Figure 2 for still frames taken from one of the videos). Ten of the videos featured people familiar to the children and 10 featured unfamiliar people. For the children with ASD, the familiar people were their intervention therapists and centre staff within their childcare playroom, and for the TD children the familiar people also comprised the staff within their childcare playroom. The criterion that was set to ensure the childcare staff was familiar to the child participants was that the children had to be enrolled for a minimum of three months in the childcare facility.

#### Video ratings

In order to ensure that the fear expressions were of the intended valence and intensity, 10 typically developing adults (4 male, mean age: 25.1) rated the neutral and fear still frames from the videos on valence and intensity using the nine-point Likert rating scales from the International Affective Picture System, Self-assessment Manikin (SAM; [80]).

#### Video selection

Three videos of familiar people (for each group) and three of unfamiliar people were selected on the basis on the mean SAM scores of valence (neutral range: 4.5 to 5.5, fear range: 1.4 to 2.7) and arousal/intensity (fear range: 5.5 to 7.6). There was a significant difference between valence ratings for neutral vs. fear (*P*s < .001), indicating that fear expressions were significantly more negative than neutral expressions. Inter-class correlations for scores were high (Cronbach’s alpha > .71) for each stimuli type (neutral, fear).

#### Video preparation

As different colours emit different levels of luminosity, the selected videos were first converted to grey scale so that pupil size would not be affected. Also, as the videos were not the same across all participants, the videos were matched on luminosity by analysis of the first still frame of the videos (per familiarity condition and group) using Adobe Photoshop 8.0 (Adobe Systems, San Jose, CA, USA). There were no significant differences in luminosity of familiar and unfamiliar stimuli, and between the stimuli for each group (all *P* > .55). A scrambled image was created from the neutral still frame for each video (and was also matched on luminosity to this still frame), which served as a buffer for the pupillary light reflex, to ensure that changes in pupil size captured during the presentation of the neutral expression were not due to pre-stimulus to stimulus luminosity changes (see Figure 2). Using iMovie HD 6.0.3 (Apple Computer Inc., Cupertino, CA, USA), the videos were adjusted so that the neutral and fearful expressions were each shown for 4 s in each video (that is, to standardise the presentation duration of expressions across the videos), and also so that the changes in pupil size could be captured to an unchanging stimulus, as subtle expression changes over time (for example, in duration of the genuine fearful expression) could account for the fluctuations in pupil size over time.

#### Everyday empathic behaviour questionnaire

The Empathy Questionnaire (EmQue) [81] was used to measure everyday empathic behaviour in the children. This parent-report questionnaire measures three traits of empathy observable in young children: 1) emotion contagion, 2) attention to others’ feelings, and 3) prosocial actions. Parents rate each item on a three-point Likert scale (0 = never, 1 = sometimes, and 2 = often), according to how applicable the behaviour has been to their child over the past two months.

### Procedure

Testing took place in a well-lit room of the childcare centre (from which the children were recruited), which had no external light. Ambient luminosity was checked before the start of each testing session using a handheld photometer (model PLMX, Quantam Instruments, Bartlett, IL, USA) and was the same for all participants. After written consent was obtained from the parent, s/he completed the EmQue. The participant was seated in a comfortable chair (see Figure 1), approximately 60 cm (36.46° visual angle) from the eye-tracking monitor. The experimenter first calibrated the child’s eye movements with the built-in five-point Tobii Studio calibration procedure, in which the children had to track a moving dot across the screen with their eyes. Following this, each child passively viewed the videos (with the order of the familiar vs. unfamiliar videos counterbalanced within each participant group), which were interspersed between the presentation of ‘filler’ stimuli (child-friendly pictures and videos) to maintain attention [82]. Filler stimuli were linked to a button-press so that the time that these stimuli appeared on screen could be adapted flexibly for each child. Each trial consisted of a scrambled image (1 s), a neutral expression (4 s), a fearful expression (4 s), and time in-between these expressions, which varied from ‘actor’ to ‘actor’ (*M* = 2.167 s, *SD* = 0.600 s). Thus, each trial was a mean of 11.167 s. With six trials per participant (three trials for the familiar condition, three trials for the unfamiliar condition), the total experiment time was 67.002 s (that is, 6 × 11.167), or 1.12 minutes + the time for filler stimuli to be shown (which, as mentioned above, differed from child to child).

### Data reduction

Pupil data, preprocessed by the Tobii system to be free of movement-artefacts (see Apparatus section), were further processed with a custom-built LabVIEW 2010 (National Instruments, Austin, TX, USA) algorithm (Beaton, 2012, unpublished). First, samples for which only one eye was tracked were eliminated (to minimize pupil size miscalculation due to head angle or ambient light exposure). Where both eyes were tracked, a mean pupil diameter across eyes was computed. Second, to cut out extreme sample-to-sample changes in pupil diameter due to partial lid closures (common in samples either side of missing data due to blinks), samples outside 2 × standard deviation of the mean rate of change (calculated for each participant) were removed. Third, gaps in data, due to blinks, were only linearly interpolated between stable data points (traces), to a maximum of 350 ms [83,84]. A trace was deemed stable if there were a minimum of 50% of the samples in 2 × total length of the gap, pre- and post-gap. This method allowed for a differential threshold for interpolation, based on gap length and the reliability of the pre-/post-gap data. In the fourth step, pupil data that were recorded whilst the participant was looking outside of the AOI of the face area were excluded to ensure only pupillary response to the face was captured.

Visual attention data (fixation counts) were extracted from Tobii Studio using a fixation filter (I-VT), using the default pre-sets (maximum gap length: 75 ms, window length: 20 ms, velocity threshold: 30 degrees per second, maximum time between fixations: 75 ms, maximum angle between fixations: 0.5 degrees), with the exception that the minimum fixation duration was set to 100 ms. This minimum fixation duration was chosen as eye-tracking data of 100 ms or more are not only more reliable than data tracked for shorter durations [85], but are also considered to be a reliable index of what elements in a scene are actually captured and processed [86].

Finally, to compute a standardised peak pupillary response per 100 ms (since eye-tracking data are arguably not stable when recorded for less than 100 ms [85]), the following formula was used:

$$a=\left(b–c\right)/c\times 100$$

where *a* = peak amplitude of pupillary response (greatest percentage change in pupil diameter from neutral to fear), *b* = mean pupil diameter during each consecutive 100 ms of the fear expressions (40 × 100 ms = 4 s total) and *c* = mean pupil diameter during the neutral expressions. Latency of peak amplitude was therefore measured per 100 ms. Thus, the percentage change from neutral to fear was calculated for every 100 ms, and the peak was recorded as *a.* For example, if *a* for one participant was 7% on one of the conditions, and this happened in the 15^th^ block of 100 ms (that is, at 1,500 ms), then this was the latency that was recorded.

## Results

Data were first analysed for skewness, kurtosis and outliers. Data were normally distributed; therefore, parametric tests were used in all analyses. For brevity, peak amplitude of pupillary response to fear expressed by familiar, and unfamiliar people, will be referred to as ‘peak amplitude to familiar fear’ and ‘peak amplitude to unfamiliar fear’, respectively, and latency of peak amplitude will be referred to as ‘peak latency’.

### Preliminary analyses

#### Cognitive ability

The relationship between cognitive ability and peak amplitude/peak latency was examined to determine whether it was necessary to control for the difference in cognitive ability between the two groups (see Table 1). The MSEL standard score (Early Learning Composite) was not related to the dependent variables in either group: peak amplitude, peak latency, fixation counts, or the EmQue scores (*P* range = .11 to .97). Thus all analyses were conducted without covarying the MSEL standard score (n.b., the results from covarying the MSEL standard score are given in the Endnotes for comparison).

#### Baseline pupil size

We also examined whether the groups were different on their resting-state pupil size (baseline pupil size whilst at rest). No group differences were found (and these results are published in a separate article [87]).

### Main analyses

#### Visual attention

**Figure 3:**
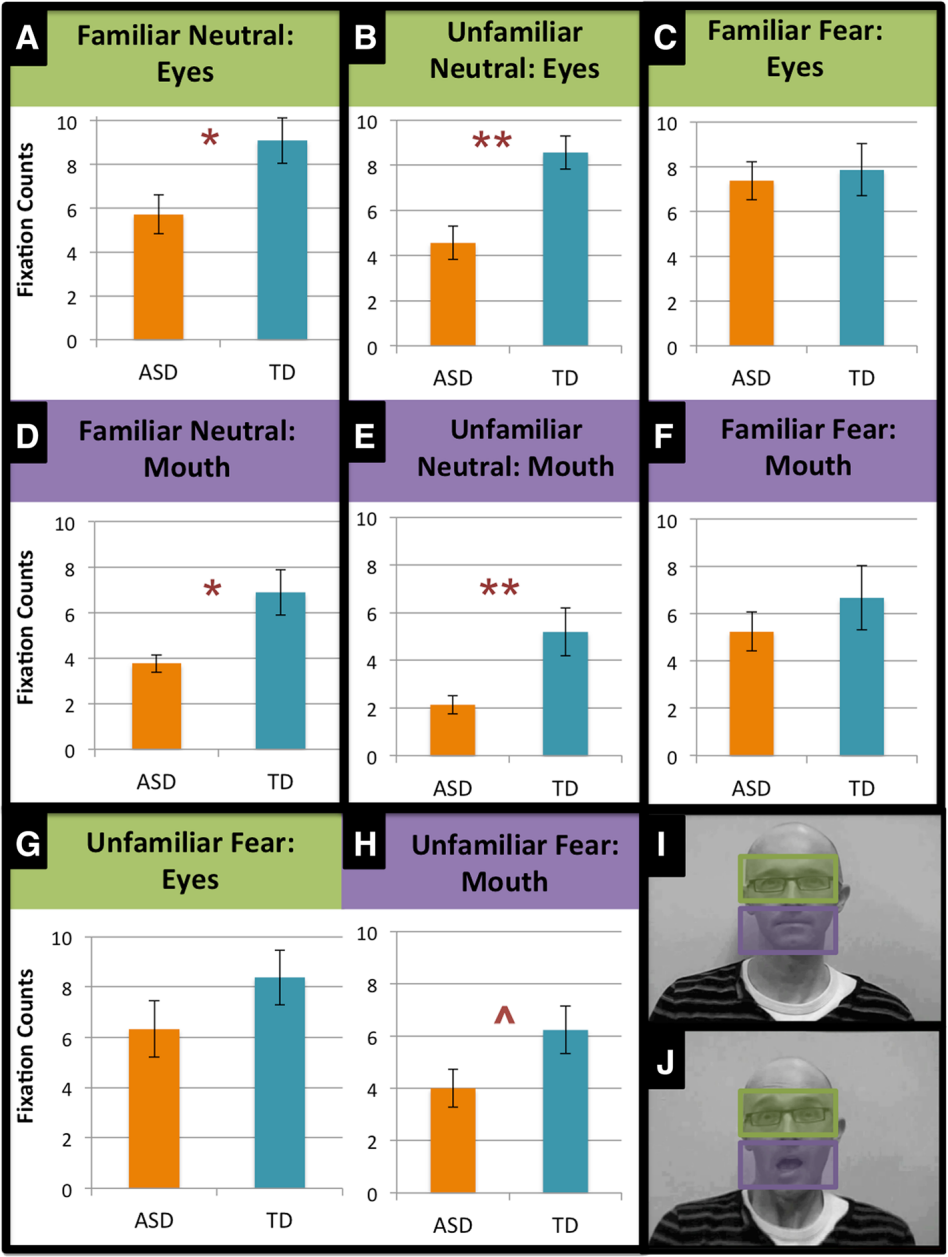
**Fixation counts (100 ms visual attention) for the facial expressions, in each condition and face area.** Bar totals represent a mean fixation count across trials. **A)**/**B)** The typically developing (TD) group looked longer at the eye region of familiar and unfamiliar people with a neutral expression than the autism spectrum disorder (ASD) group. **C)**/**G)** However, the groups were no different in visual attention to the eye region of familiar and unfamiliar people with a fearful expression. **D)**/**E)** Likewise, the TD group looked longer than the ASD group to the mouth region of familiar and unfamiliar people with a neutral expression. **F)**/**H)** Similarly, the groups were no different in visual attention to the mouth region of familiar people with a fearful expression, though the groups were marginally different on visual attention to the mouth region of unfamiliar people with a fearful expression, with the TD group looking longer than the ASD group. **I)**/**J)** Mouth and eye region areas of interest (AOIs). ^*P* < .10, *P < .05, **P < .01. ^e^

To determine whether the groups differed on their visual attention to the faces, a four-way ANOVA (two Groups × two Familiarity levels × two Emotions × two Face areas) was conducted on the fixation counts (100 ms visual attention). The main effect of Familiarity was significant, *F*(1,40) = 8.86, *P =* .005, *η*^*2*^ *=* .18, as was the main effect of AOI, *F*(1,40) = 9.67, *P =* .003, *η*^*2*^ *=* .20. The main effects of Group and Emotion were also significant, *F*(1,40) = 9.31, *P =* .004, *η*^*2*^ *=* .19, and *F*(1,40) = 4.59, *P =* .04, *η*^*2*^ *=* .10, respectively, which were driven by the significant Group × Emotion interaction, *F*(1,40) = 6.23, *P =* .01, *η*^*2*^ *=* .14. Pairwise comparisons showed that there was no difference between the groups on fixation counts to the faces expressing fear (*P =* .12, *η*^*2*^ *=* .06), but the TD group looked more to the neutral expressions than did the ASD group (*P <* .001, *η*^*2*^ *=* .31). Further, although the TD group did not differentiate their visual attention between neutral and fear, the ASD looked longer at the fearful expressions compared to the neutral expressions (*P =* .002, *η*^*2*^ *=* .22). ^a^ No other interaction effects were significant. Means are displayed in Figure 3 (for comparison, significant independent samples *t*-tests are also marked here). To minimise potential effects of group differences in visual attention, fixation count was covaried in subsequent analyses, using a composite score of fixation counts to the face area across all conditions. The total number of fixation counts to the whole Face AOI across experimental conditions (familiarity, emotion) for the groups (averaged across trials) was *M =* 44.19 (4.419 secs), *SD =* 20.70 for the ASD group and *M =* 58.95 (5.895 secs), *SD =* 23.98 for the TD group.

#### Peak amplitude

**Figure 4:**
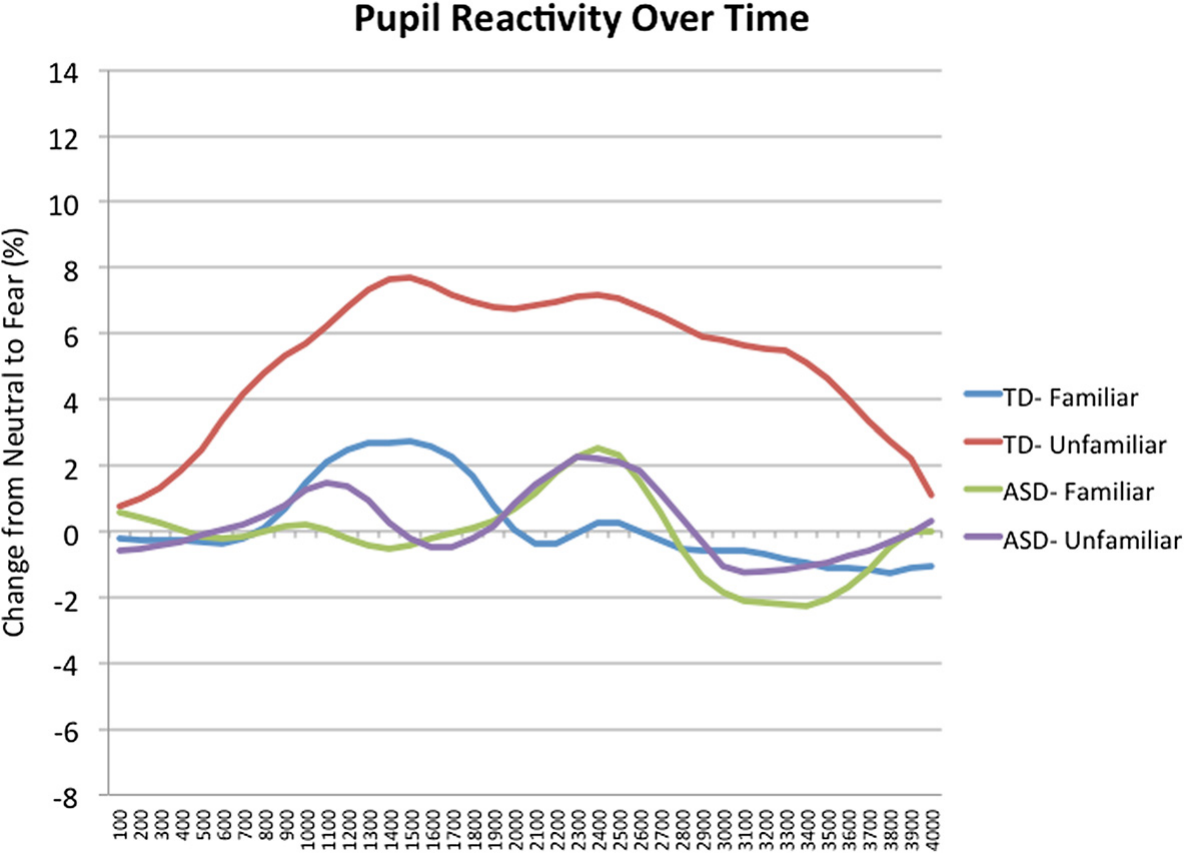
**Change in pupil diameter from neutral expression to fear expression over time (shown as a percentage).** Unadjusted means, in increments of 100 ms and smoothed with a seven-point moving average filter, for each familiarity condition and group.

**Figure 5:**
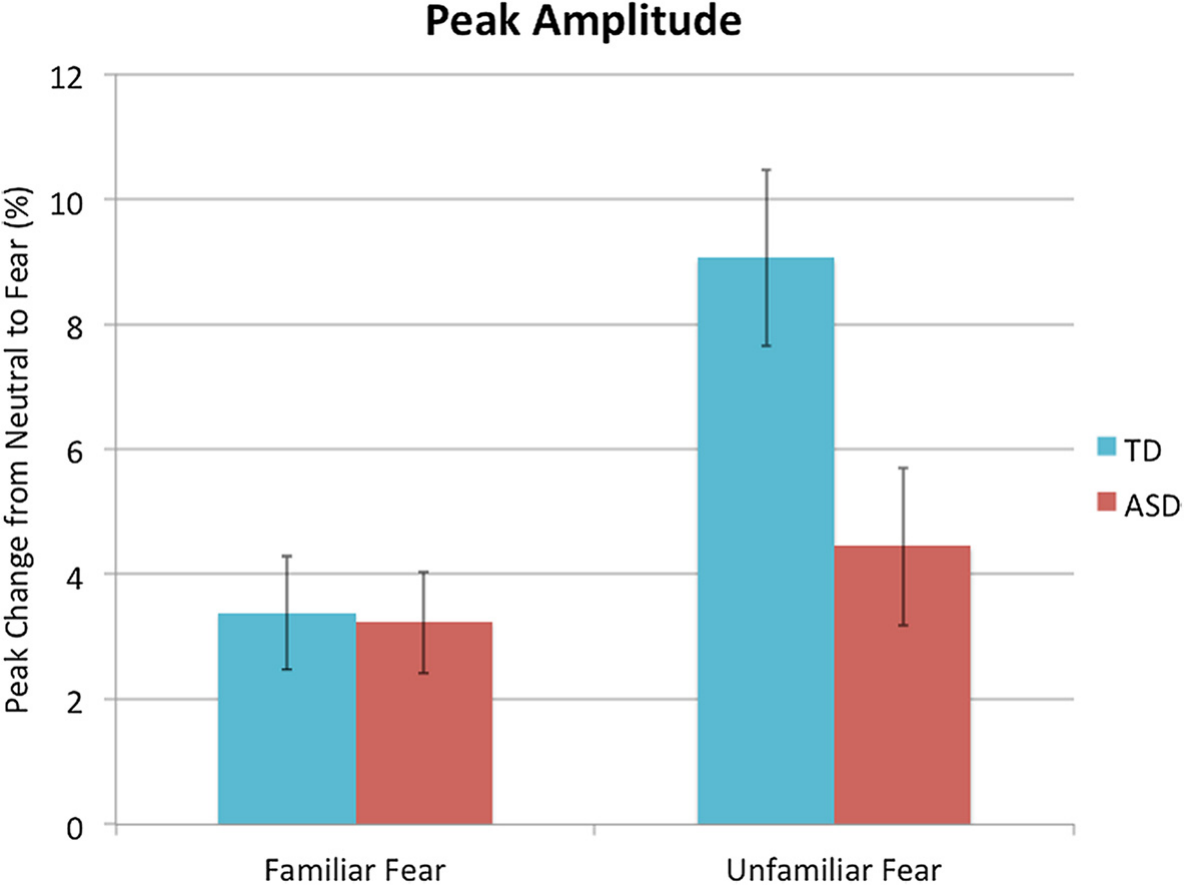
**Peak change in pupil diameter from neutral expression to fear expression (shown as a percentage).** Unadjusted means for each familiarity condition is pictured here, for each group. Error bars represent standard error.

To determine whether the groups differed on reactivity to fear in familiar and unfamiliar people, a two-way ANCOVA (two Groups × two Familiarity levels) was conducted on the peak amplitude (change from neutral to fear expression), controlling for visual attention (fixation counts) to the stimuli. ^b ^Percentage change, from the neutral to fearful expression, over time is pictured in Figure 4. The main effect of Familiarity was not significant, *F*(1,39) = 1.80, *P =* .19, *η*^*2*^ *=* .04, but there was a main effect of Group, *F*(1,39) = 6.64, *P =* .01, *η*^*2*^ *=* .15, which was driven by the significant Group × Familiarity interaction, *F*(1,39) = 6.25, *P =* .02, *η*^*2*^ *=* .14. The effect of the visual attention covariate was not significant, *F*(1,39) = 0.26, *P =* .61, *η*^*2*^ *=* .007, therefore, the unadjusted mean scores are shown in Figure 5. Pairwise comparisons showed that there was no difference between the groups on peak amplitude to familiar fear (*P =* .67, *η*^*2*^ *=* .005), but the ASD group had reduced peak amplitude to unfamiliar fear (*P =* .005, *η*^*2*^ *=* .18) compared to the TD group, who had a greater peak amplitude to unfamiliar fear than to familiar fear (*P =* .002, *η*^*2*^ *=* .22); the ASD group did not differentiate between familiarity conditions (*P =* .78, *η*^*2*^ *=* .002). ^c^

#### Peak latency

**Figure 6:**
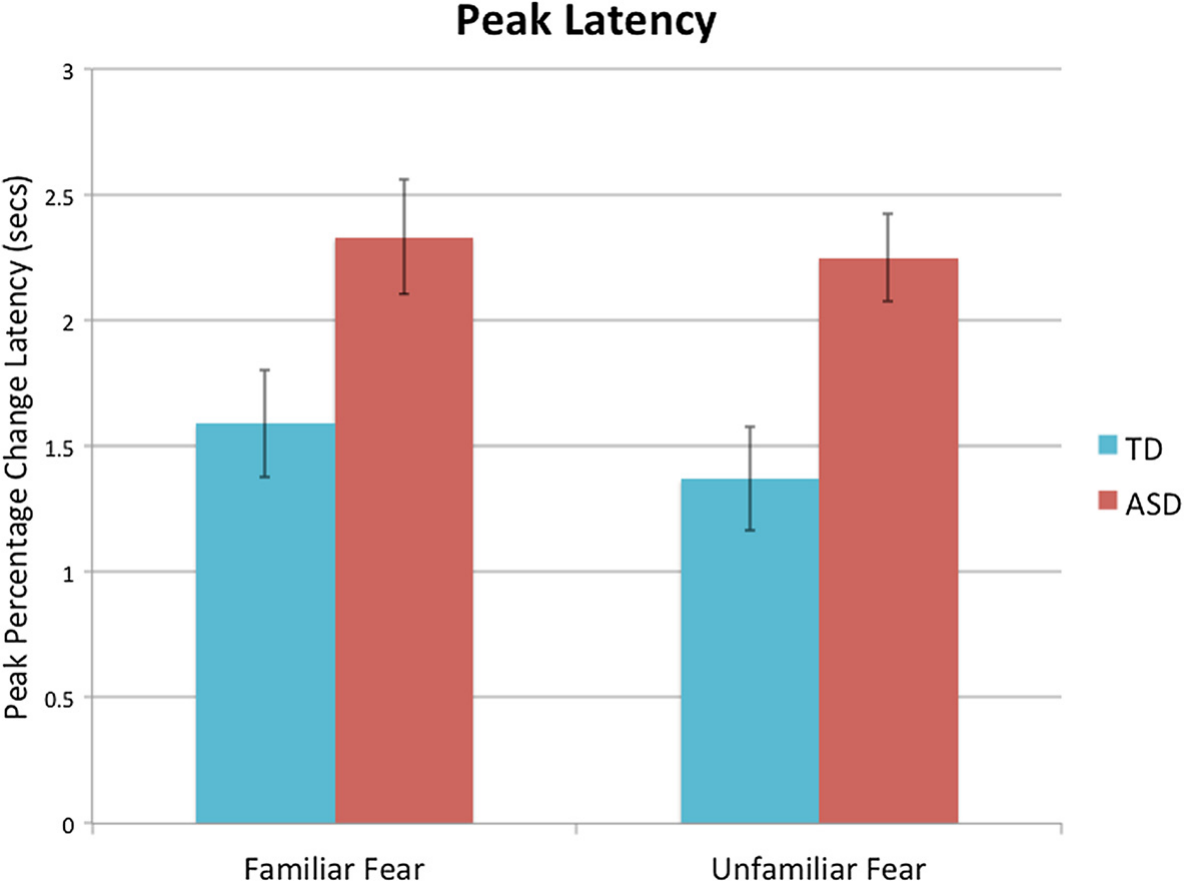
**Peak pupillary change latency, in seconds, during the fear expression.** Unadjusted means for each group and familiarity condition is shown. Error bars represent standard error.

To determine whether the groups differed in peak latency, a two-way ANCOVA (two Groups × two Familiarity levels), again controlling for visual attention, was performed on peak latency (latency to peak amplitude change from neutral to fear expression). As is evident in Figure 6, there was a significant main effect of Group, *F*(1,39) = 14.53, *P <* .001, *η*^*2*^ *=* .27, but no main effect of Familiarity, *F*(1,39) = .34, *P =* .57, *η*^*2*^ *=* .009, nor a Group × Familiarity interaction, *F*(1,39) < .001, *P =* .998, *η*^*2*^ *<* .001, indicating that the children with ASD showed a longer response latency, compared to the TD group, across the familiarity conditions. The effect of the visual attention covariate was not significant *F*(1,39) = 0.88, *P =* .36, *η*^*2*^ *<* .02. ^d^

#### Everyday empathic behaviour

To determine whether the groups differed in everyday empathic behaviour, *t-*tests were computed on the difference between the scores for each group on the EmQue sub-scales, and the EmQue total score. As can be seen in Table 2, children in the ASD group scored significantly lower on the EmQue total score and on the Attention to Others’ Feeling and Prosocial Behaviour EmQue sub-scales. Although the *t*-test did not differentiate the groups on the EmQue Emotional Contagion sub-scale, the ASD group had lower scores, and there was a medium effect size of the difference between the groups [88].

**Table 2:** Group differences on everyday empathic behaviour, as measured by the EmQue

	***ASD group*** **M (SD)**	***TD group*** **M (SD)**	***t-*** **value**	***P-*** **value**	***Cohen’s d***
EmQue TOTAL	11.95 (7.16)	21.33 (4.71)	4.99	<.00	1.60
†Emotional contagion	2.65 (2.46)	3.81 (2.21)	1.59	.12	.51
†#Attention	6.60 (3.49)	10.76 (1.92)	4.77	<.00	1.53
†Prosocial behaviour	2.70 (2.98)	6.76 (1.81)	5.31	<.00	1.70

†EmQue sub-scales, #Attention to Others’ Feelings.

### Correlation analyses

#### Associations with visual attention

To determine whether emotional reactivity was associated with visual attention, Pearson correlations were computed between fixation counts to the eye, mouth and face areas (eye + mouth AOI) and peak amplitude/peak latency (within each familiarity condition). As apparent in Table 3, in the ASD group, peak amplitude to familiar fear was positively related to fixation counts to the fearful familiar face (*r* = .51; *P* = .02), which was driven by fixation counts to the eye region of familiar faces (*r* = .64; *P* = .002). In the TD group, however, peak amplitude to familiar fear was positively related to fixation counts to the mouth region of familiar faces (*r* = .44; *P* = .045). Moreover, in the TD group, an association was also found between peak latency to familiar fear and fixation counts to fearful familiar faces (*r* = .43; *P* = .051), which appeared to be driven by fixation counts to the mouth region of familiar faces (*r* = .38; *P* = .09). No other correlations between fixations counts and peak amplitude/peak latency for either group were significant (all *P*s = .14 to .998).

**Table 3:** Correlations between visual attention and emotional reactivity

	***Familiar***		***Unfamiliar***	
	***Peak amplitude***	***Peak latency***	***Peak amplitude***	***Peak latency***
*ASD group*				
Neutral face	.28 (.18)	-.08 (-.07)	.20 (.07)	.05 (.24)
Neutral eyes	.24 (.19)	-.09 (-.08)	-.08 (-.17)	.04 (.12)
Neutral mouth	.13 (-.007)	.002 (.02)	.32 (.29)	.17 (.21)
Fearful face	.51* (.44*)	-.17 (-.17)	.18 (.13)	-.07 (-.02)
Fearful eyes	.64** (.60**)	-.34 (-.34)	.07 (-.01)	-.21 (-.16)
Fearful mouth	.07 (-.001)	.10 (.11)	.27 (.28)	.20 (.21)
*TD group*				
Neutral face	.12 (.10)	.17 (.11)	-.002 (-.005)	.25 (.26)
Neutral eyes	-.23 (-.23)	.09 (.08)	-.18 (-.18)	.33 (.32)
Neutral mouth	.32 (.32)	.14 (.06)	.11 (.10)	.05 (.07)
Fearful face	.23 (.22)	.43^ (.37)	.04 (.03)	-.17 (-.16)
Fearful eyes	-.16 (-.19)	.23 (.17)	-.14 (-.15)	-.31 (-.30)
Fearful mouth	.44* (.44*)	.38^ (.33)	.24 (.23)	.09 (.10)

The visual attention variable was Fixation Counts (100 ms visual attention). Each Fixation Count variable is only correlated with the corresponding familiarity level of Peak Amplitude/Peak Latency (for example, the correlation between Unfamiliar Peak Amplitude and Fear FACE is only Fear FACE on the Unfamiliar condition (not a composite score of the Unfamiliar and Familiar conditions). Face area of interest (AOI) = eye AOI + mouth AOI. The table shows Pearson correlations (two-tailed) between visual attention (fixation counts) and emotional reactivity, as indexed by peak amplitude and peak latency. The r-values for partial correlations, controlling for the effect of cognitive ability (MSEL standard score), are in parentheses, and r-values outside parentheses are without controlling for this effect. Significant correlations (α = .05) are in bold type. **P < .01, *P < .05, *^P* < .10.

#### Associations with empathic behaviour

To explore the relationships between emotional reactivity and everyday empathic behaviour, we computed Pearson correlations between the emotion variables (peak amplitude, peak latency) for each condition and EmQue scores. These correlations, both with and without partialling out the effect of cognitive ability (MSEL standard score) are shown in Table 4. The overall pattern of correlations is not affected by partialling out the effect of cognitive ability.

**Table 4:** Correlations between empathic behaviour and emotional reactivity

	***Peak amplitude***		***Peak latency***	
	***Familiar***	***Unfamiliar***	***Familiar***	***Unfamiliar***
*ASD group*				
EmQue total	.06 (.03)	-.09 (-.11)	-.20 (-.19)	-.20 (-.19)
†Emotional contagion	-.33 (-.29)	-.20 (-.17)	-.11 (-.12)	.43 (.41)
†#Attention	.18 (.12)	-.07 (-.11)	-.17 (-.17)	-.25 (-.22)
†Prosocial behaviour	.21 (.17)	-.03 (.004)	-.18 (-.18)	-.55* (-.53*)
*TD group*				
EmQue total	-.14 (-.15)	-.34 (-.35)	.16 (.16)	-.29 (-.29)
†Emotional contagion	.12 (.12)	-.28 (-.29)	.28 (.26)	-.33 (-.32)
†#Attention	-.19 (-.19)	-.44* (-.44*)	-.004 (.000)	-.06 (-.06)
†Prosocial behaviour	-.32 (-.33)	-.08 (-.08)	.09 (.09)	-.30 (-.30)

This table shows the Pearson correlations (two-tailed) between everyday empathic behaviour (EmQue scores) and emotional reactivity as indexed by peak amplitude (familiar, unfamiliar) and peak latency (familiar, unfamiliar, across familiarity conditions). The r-values for partial correlations, controlling for the effect of cognitive ability (MSEL standard score), are in parentheses, and r-values outside parentheses are without controlling for this effect. † EmQue sub-scales, # Attention to Others’ Feelings, *P ≤ .05. Significant correlations are in bold.

In the ASD group, the Prosocial Behaviour sub-scale of the EmQue was negatively related with peak latency to unfamiliar fear (*r* = -.55, *P* = .01), suggesting that the children with ASD who react more quickly to the fearful expressions of unfamiliar people, are more pro-social. In the TD group, the Attention to Others’ Feelings sub-scale was negatively related to peak amplitude to unfamiliar fear (*r* = -.44, *P* = .045), indicating that the TD children who responded less to the fearful expressions of unfamiliar people are reported as being more attentive to others’ feelings.

#### Associations with autistic symptoms

To determine whether autistic symptoms were related to pupillary reactivity, Pearson correlations between ADOS algorithm scores and peak amplitude/latency were computed for the ASD group. The Communication Algorithm and the Play Algorithm of the ADOS were both moderately negatively associated with peak amplitude of response to familiar fear (*r* = -.42, *P* = .06, and *r* = -.39, *P* = .08, respectively), but not with peak amplitude to unfamiliar fear (*P*s > .35). The Social Algorithm and the Restrictive and Repetitive Behaviours Algorithm were not related to any of these dependent variables (all *P*s > .18).

## Discussion

The main aim of the current study was to establish whether children with ASD react differentially to emotion in familiar vs. unfamiliar people, and whether they differ from typically developing children in their reactivity. In terms of amplitude of emotional response, as expected, the children with ASD reacted normatively to the fearful expressions of familiar people, which is consistent with previous findings on brain activity to familiar neutral faces in ASD [36-38]. However, the children with ASD were found to have reduced pupillary responses to the fearful expressions of unfamiliar people, relative to the typically developing children, which is also consistent with numerous previous reports mentioned above [4]. This pattern of findings was independent of visual attention (group differences were based on emotion, not familiarity) or cognitive factors, indicating a difference in emotional reactivity to unfamiliar people *per se*. Thus, the data indicate that the often-reported abnormalities in emotional responses in this population may be particular to the emotions of unfamiliar people. It may be that for the children with ASD, familiarity with a person is necessary for an emotional response, which is not the pattern seen in typical development. These findings suggest that the neural architecture for emotion processing may be functional in ASD, but may require bootstrapping from circuits involved with the processing of familiar persons. For instance, it may be that the extraction of emotional information from faces, or the motivational drive to do so, is generally disrupted in ASD, but that this disruption may not apply to familiar people, that is, individuals with ASD might be more motivated to extract this information from people they know. This may be based not only on their affiliative bonds with familiar others, but also, perhaps as a consequence of, the more frequent emotional learning opportunities that come with familiar others. Thus, it may be that the emotional learning that derives from familiar people may not generalize to the emotions of unfamiliar people. Indeed, this interpretation is consistent with the often-reported difficulty in ASD with the generalization of skills across contexts (for example, [89]). Whereas in typical development this explicit-level reasoning does not seem related to emotional reactivity (as it seems to be based on implicit circuits), in ASD, emotional reactivity may rely on explicit emotion processing strategies [4,90,91]. Thus, it may be that explicit processing of the personal relevance of others’ emotions may help to bootstrap underpowered emotional brain circuits in individuals with ASD.

Many studies have identified reduced amygdala activation in individuals with ASD during the processing of emotions [90,92,93], and it is well established that the amygdala is a key structure in fear responses [94-96]. Both neuroanatomical and neuroimaging data suggest that pupillary responses are functionally linked to the amygdala [97-101]. Thus, the pupillary results presented in this study are consistent with the findings of Pierce and colleagues [36-38], who found a normative amygdala response in individuals with ASD to familiar, but not unfamiliar (neutral) faces. Together, these results may suggest that when individuals with ASD process the fearful expressions of familiar persons, this processing is at least partially mediated by the amygdala. However, the fearful expressions of unfamiliar people do not seem to engage the amygdala in the same way in these individuals. As pupillary responses are also mediated by the locus coeruleus [102-104], these results are also consistent with previous literature indicating abnormalities in the locus coeruleus-noradrenergic brain system which underlies physiological responses to stress in individuals with ASD [105,106].

The findings relating to visual attention showed that compared to the TD children, the children with ASD had reduced visual attention to the neutral, but not the fearful expressions. Further, whereas the TD group did not differentiate between neutral and fearful expressions in their visual attention, the children with ASD did, with more visual attention towards the fearful expressions compared to the neutral expressions. This result seems to suggest that the visual attention of children with ASD is not ‘captured’ by neutrally expressive faces, as it is in typical development, but emotionally expressive faces help to close this attention gap between the groups. This pattern of results was also found in an eye-tracking study by Vivanti and colleagues [107], and is consistent with many studies showing less attention to (neutral) faces in ASD [108,109].

Interestingly, the visual attention of the children with ASD to the eye region of fearful familiar faces was related to their pupillary response amplitude to these expressions. As this relationship was not found in both familiarity conditions, nor with both emotions (that is, neutral, fear), there does not appear to be an overall hyper-arousal to the eye region as suggested by some scholars [39,110]. Moreover, for the fearful expressions of familiar people, the peak amplitude and number of fixations to the eye region were normative in the ASD group, suggesting that this relationship is mediated by more ‘normative’ scanning patterns, that is, the children with ASD did look at the eye region and, as a consequence, had normative reactivity to fearful expressions of familiar people. However, attention was not associated with the reduced response to emotion in unfamiliar people in the ASD group, and domain-general factors other than attention, such as motivation or perception, as mentioned above, are likely to play a role [4].

Although the amplitude of the pupillary response to fearful expressions of familiar people was similar across groups, the children with ASD showed longer latency responses in both familiarity conditions. Thus, children with ASD seem to have a general slowing in emotional responses, which is consistent with research showing longer latencies in emotion recognition, facial reactions and ERPs to emotions [50,48,51], but not with initial findings of response latency to familiar person stimuli [54]. Thus, regardless of whether the fearful expression was by familiar or unfamiliar people, the data suggest that children with ASD have longer latency emotional responses. This finding is consistent with self-reports of individuals with ASD, one person describing that *“…*there are the times when it all feels like an intricate dance*,* and I’m a step out of synch with everyone else around me” [111]. Some scholars have even suggested that emotional communication difficulties in ASD, along with other symptoms of this disorder, stem from a general problem with temporo-spatial processing (for review, see [112]). Others have suggested that temporal processing delays in ASD are related to less strengthened long-range connections between sub-cortical and cortical areas [113]. Indeed, some functional connectivity studies in ASD have documented less coupling between the amygdala and the superior temporal sulcus, pre-frontal cortex, temporal lobe, premotor cortex and inferior frontal gyrus, and between the amygdala and fusiform via the primary visual cortex, during emotion processing [114-116]. Moreover, some evidence suggests that abnormalities in synaptic homeostasis are a risk factor for ASD [117]. Thus, it is possible that our finding of a general delay in emotional responsivity in the ASD group may be reflective of delays or abnormalities in neural connectivity in this population. In the context of a fast-paced social interaction, slow emotional responses are likely to impact upon communication and social-reciprocity for children with ASD, and their construction of meaning of inter-personal communicative exchanges would consequently be disrupted.

The latency of emotional response amongst the children with ASD, particularly to unfamiliar people, appears to play an important role in their everyday emotional behaviour, as it was found that those children whose parents rated them as more prosocial have faster emotional responses. Thus, acting prosocially is related to the speed of reacting emotionally, which is consistent with previous reports of a relationship between ERP latency to emotional expressions with empathic behaviour [52] and emotion recognition [53].

Emotional reactivity also seems to have an association with ASD symptoms, with larger amplitude responses to fearful expressions of familiar people being related to fewer communication and play deficits. An important area for future research would be to examine the inter-relationship between emotional responses to familiar others and communication/play in children with ASD over the first years of life. One may posit that a greater response to the emotions of familiar others is a protective factor for the communication and play deficits associated with ASD. Given the interplay of emotion and social factors, that are both affected in ASD, a longitudinal study is necessary to establish the causal directions between such factors. By examining children in the first and second year of life, it could be established whether there are any critical periods for social communicative development which are related to response to emotion in familiar people.

### Limitations

Some limitations in the current study should be noted. Firstly, while the inclusion of an age-matched typically developing group was very useful to understand normative reactivity, the two groups were not matched on cognitive ability. The inclusion of a chronological and mental age-matched group would be ideal, and further research should incorporate such a control group. However, it should be noted that the dependent variables were not related to cognitive ability and even when cognitive ability was controlled, the pattern of results for each analysis remained largely unchanged (see Endnotes).

Secondly, following the recommendations of Nakagawa and Perneger [118,119], due to our small sample sizes, we did not correct for multiple comparisons as traditional methods tend to be overly conservative, and we wanted to avoid inflating the probability of Type-II errors. Nevertheless, this must be taken into account when considering the results from the current study.

Thirdly, although research has indicated that pupil size is related to emotional arousal, for example, [55] (that is, more sympathetic ANS activity) in typically developing adults, more research is needed to establish whether the same physiological processes mediate emotional responses in children (with and without autism), as pupil dilation may also be related to less parasympathetic ANS activity in response to emotions in these populations.

Lastly, the analyses presented above were based on relatively small amounts of data (an average of less than two seconds in each condition, for each group, across only three trials). To avoid this issue, future research should include more trials of each familiarity condition; in the current study this was limited by the availability of familiar people to the children in both groups who made easily recognisable fearful facial expressions. Nevertheless, previous research suggests that conscious perception of emotional facial expressions is quite rapid (from 100 to 150 ms; [120]), thus the current results may not be affected.

## Conclusions

Overall, the findings suggest that emotional reactivity in children with ASD to fearful expressions of familiar people is similar in magnitude, but slower than typically developing children, and this delayed response appears to be related to their prosocial behaviour. On the contrary, reactivity to fear expressed by unfamiliar people was abnormal in the ASD group in terms of both magnitude and latency. This pattern of results suggests that emotion processing difficulties in ASD appears not be an absolute impairment, but rather one that may be mediated by familiarity.

## Endnotes

^a^When also controlling for cognitive ability, the only significant effect was that of Group, *F*(1,39) = 4.34, *P =* .044, *η*^*2*^ *=* .10. However, the cognitive ability covariate was not significant *F*(1,39) = 0.16, *P =* .69, *η*^*2*^ *=* .004.

**Figure 7:**
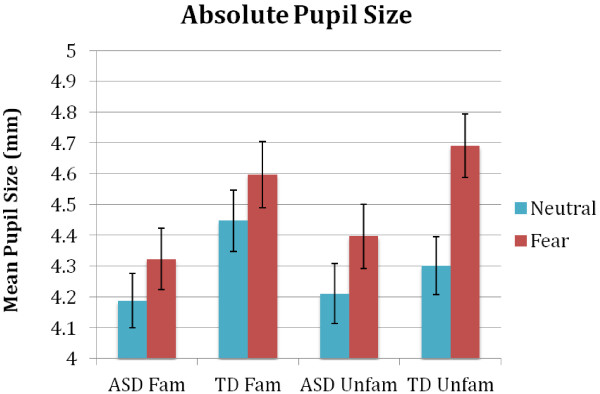
Mean absolute pupil sizes on each condition, for each group.

^c^When also controlling for cognitive ability the same overall pattern of results emerges. Pairwise comparisons show that the Group × Familiarity interaction, *F*(1,38) = 4.10, *P =* .05, *η*^*2*^ *=* .10, is again driven by no group difference on peak amplitude to familiar fear (*P =* .48, *η*^*2*^ *=* .01) but a reduced peak amplitude to unfamiliar fear in the ASD group compared to the TD group (*P =* .04, *η*^*2*^ *=* .11); in addition, there is a greater peak amplitude response to unfamiliar compared to familiar fear, which again can be seen in the TD group (*P =* .005, *η*^*2*^ *=* .19), but not in the ASD group (*P =* .78, *η*^*2*^ *=* .002). The effect of the cognitive ability covariate was not significant *F*(1,38) = 0.004, *P =* .95, *η*^*2*^ *<* .001.

^d^When also controlling for cognitive ability, again, the same pattern of results emerges; the only significant effect was that of Group, *F*(1,38) = 6.56, *P =* .02, *η*^*2*^ *=* .15. The cognitive ability covariate was not significant *F*(1,38) = 0.19, *P =* .66, *η*^*2*^ *=* .005.

^e^Written consent was obtained from the people featured in Figures 2 and 3 for the publication of their images.

^b^To aid with interpretation of the pupillary change score data, the mean absolute pupil sizes on each of the two familiarity conditions for neutral and fear are also reported below (Fam = familiar condition, Unfam = unfamiliar condition) (Figure 7).

## Abbreviations

ADOS: Autism diagnostic observation schedule; ANS: Autonomic nervous system; AOI: Area of interest; ASD: Autism spectrum disorder; EmQue: Empathy Questionnaire; ERPs: Event-related potentials; MSEL: Mullen scales of early learning; RSA: Respiratory sinus arrhythmia; SAM: Self-assessment Manikin; TD: Typically developing.

## Competing interests

The authors declare that they have no competing interests.

## Authors’ contributions

HN, GV and CD conceived of the study and design. HN conducted the research testing, arranged for the pupillary data reduction and analysed the data with guidance from GV and CD. HN and GV were involved in the interpretation of the data. HN drafted the manuscript, and GV and CD revised it critically, giving important intellectual content. All authors read and approved the final manuscript.
